# Assay development and inhibition of the *Mt*-DprE2 essential reductase from *Mycobacterium tuberculosis*

**DOI:** 10.1099/mic.0.001288

**Published:** 2023-01-23

**Authors:** Sarah M. Batt, Szilvi Toth, Beatriz Rodriguez, Katherine A. Abrahams, Natacha Veerapen, Giacomo Chiodarelli, Liam R. Cox, Patrick J. Moynihan, Joel Lelievre, Klaus Fütterer, Gurdyal S. Besra

**Affiliations:** ^1^​ Institute of Microbiology and Infection, School of Biosciences, University of Birmingham, Birmingham B15 2TT, UK; ^2^​ Diseases of the Developing World, GlaxoSmithKline, Severo Ochoa 2, 28760, Tres Cantos, Madrid, Spain; ^3^​ School of Chemistry, University of Birmingham, Edgbaston, Birmingham, UK

**Keywords:** decaprenylphosphoryl-D-arabinose, enzyme kinetics, *Mt*-DprE1, *Mt*-DprE2, *Mycobacterium tuberculosis*, mycobacterial cell wall, substrate inhibition

## Abstract

DprE2 is an essential enzyme in the synthesis of decaprenylphosphoryl-β-d-arabinofuranose (DPA) and subsequently arabinogalactan, and is a significant new drug target for *M. tuberculosis *. Two compounds from the GSK-177 box set, GSK301A and GSK032A, were identified through *Mt*-DprE2-target overexpression studies. The *Mt*-DprE1-DprE2 complex was co-purified and a new *in vitro* DprE2 assay developed, based on the oxidation of the reduced nicotinamide adenine dinucleotide cofactor of DprE2 (NADH/NADPH). The *Mt*-DprE1-DprE2 complex showed interesting kinetics in both the DprE1 resazurin-based assay, where *Mt*-DprE2 was found to enhance *Mt*-DprE1 activity and reduce substrate inhibition; and also in the DprE2 assay, which similarly exhibited substrate inhibition and a difference in kinetics of the two potential cofactors, NADH and NADPH. Although, no inhibition was observed in the DprE2 assay by the two GSK set compounds, spontaneous mutant generation indicated a possible explanation in the form of a pro-drug activation pathway, involving *fgd1* and *fbiC*.

## Introduction

*Mycobacterium tuberculosis* (*Mtb*), the causative agent of tuberculosis, was responsible for 1.4 million fatalities in 2019 [1]. Many of the current first- and second-line drugs have been in circulation for over 50 years, with resistant strains rife, in the form of MDR (multidrug-resistant) and XDR (extremely drug-resistant) strains [2]. Consequently, research into drug development and target validation has exploded in recent years [3]. Drug libraries from synthetic and natural products from a variety of sources are often used to initially identify high potency inhibitors [4, 5], which can rapidly isolate new drug targets through a combination of approaches using the generation of spontaneous resistant mutants and whole-genome sequencing, over-expression studies in whole-cell assays, chemical proteomics, structural studies and binding assays [3]. In addition, structural studies of targets and inhibitor complexes enables further hit and lead optimization for more favourable properties [6–8].

**Fig. 1.**
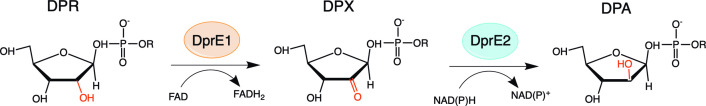
Schematic representation of the epimerase reaction catalysed by the *Mt*-DprE1-DprE2 complex. R=decaprenyl chain.

Decaprenylphosphoryl-β-d-arabinofuranose (DPA) is a membrane-anchored substrate utilized by all cell-wall arabinosyltransferases and is essential for the synthesis of the arabinan-containing layers of both cell-wall arabinogalactan and lipoarabinomannan [9–12]. Catalysis of the final step of DPA synthesis occurs through DprE1 (Rv3790, decaprenylphosphoryl-β-d-ribose oxidase) and DprE2 (Rv3791, decaprenylphosphoryl-2-keto-β-d-erythro-pentose reductase), which epimerise the ribose of decaprenylphosphoryl-β-d-ribose (DPR) to arabinose (furanose form) of DPA (Fig. 1). Flavin adenine dinucleotide (FAD)-containing DprE1 performs the initial oxidation of the C-2 hydroxyl group to a keto-intermediate, DPX (decaprenylphosphoryl-β-d-2′-keto-*erythro*-pentafuranose), which is then reduced back to a hydroxyl group by DprE2, using the reduced form of the nicotinamide-adenine dinucleotide cofactor (NADPH or NADH) [11]. Both DprE1 and DprE2 have been shown to be essential for viability [13–15].

DprE1 is the target of the nitro-benzothiazinones (BTZ) class of drugs, which show extremely low MICs and low toxicity [16, 17]. The most promising, BTZ043 and Macozinone (PBTZ169), are currently in phase I clinical trials, with MICs of 1 ng ml^−1^ and 0.3 ng ml^−1^ against *Mtb*, respectively [16, 18, 19]. Consequently, DprE1 has been the focus of much research, including structural studies [20], high-throughput target-based screening [21] and structure-based design [22–24]. The resulting plethora of new DprE1 inhibitors, includes TCA1 [25], GSK710 [21] and azaindoles [26], all of which bind non-covalently in a mechanism that differs from that of BTZ and PBTZ169.

However, much less is known of DprE2, the second essential enzyme in the epimerization reaction of DPR to DPA. In this study, we have identified a series of inhibitors that target *Mt*-DprE2 using whole-cell target overexpression studies and purified the protein to homogeneity, in complex with *Mt*-DprE1, for subsequent assay development and structural studies.

## Results

### *Mt*-DprE2 is the target of compounds from the GSK TB-set 177 library

A previous screening campaign had identified a set of 177 compounds that showed high potency in suppressing growth of *Mtb* in liquid culture, combined with non-toxicity in mammalian cell lines [4].

To assess whether the 177 Box set included compounds that target *Mt*-DprE2, a whole-cell target overexpression study directed at *Mt*-DprE2 was carried out using the vaccine strain *Mycobacterium bovis* BCG, monitoring increases in the MIC of the compounds, as previously described for *Mt*-DprE1 [21]. The gene *Mt-dprE2* (Rv3791) was cloned into plasmid pMV261, to generate pMV261-*Mt-dprE2*, a multicopy plasmid that expresses *Mt-*DprE2 under the control of a constitutive promoter in *M. bovis* BCG.

**Fig. 2.**
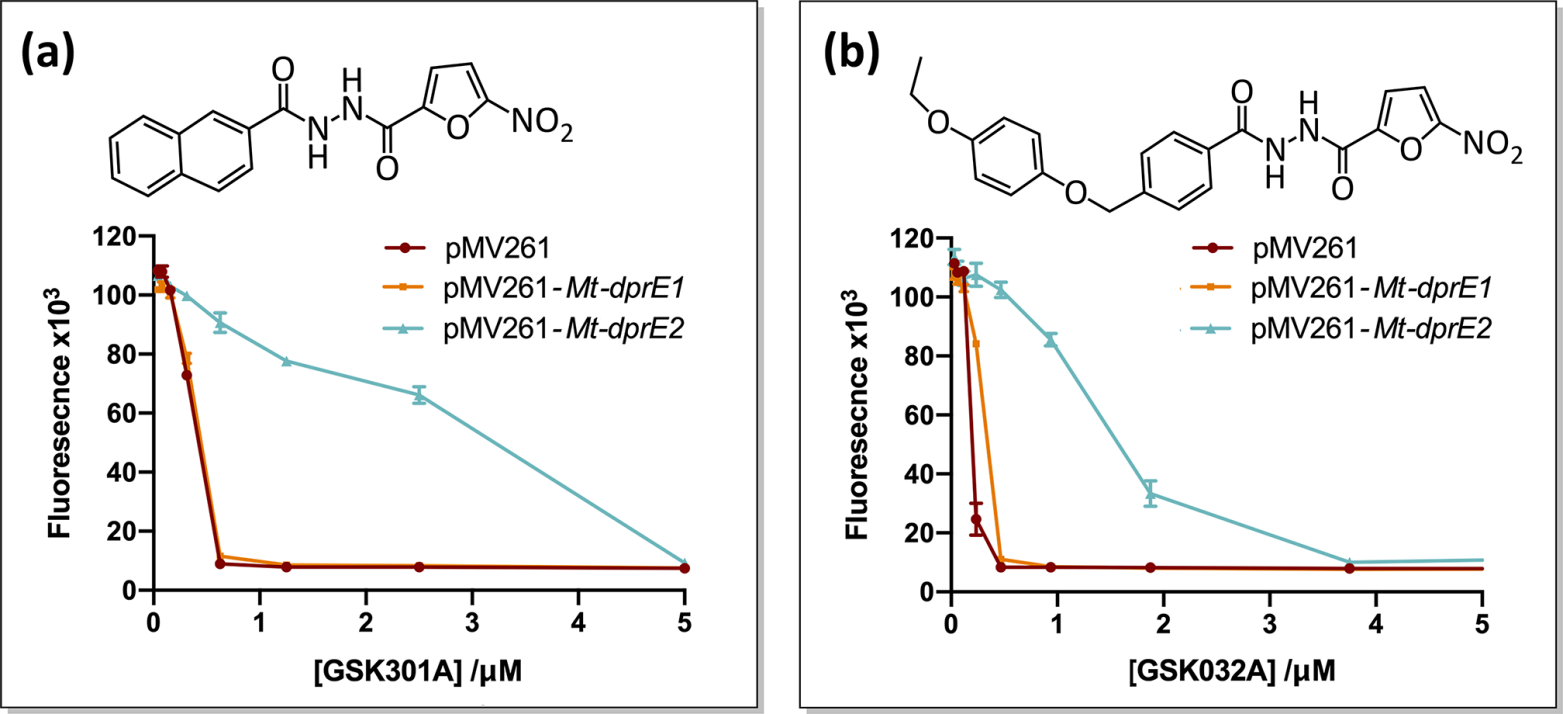
Target overexpression MIC analysis. Overexpression of *Mt*-DprE2 increases the MIC of *M. bovis* BCG for both, (a) GSK301A and (b) GSK032A, by eightfold. Survival was measured using a plate-based resazurin assay, which is reduced to the fluorescent resorufin product by living cells.

Initially, the GSK TB-set was screened at 2 µM and 20 µM, to ensure maximum coverage of the MICs of the different compounds. We compared growth of the *M. bovis* BCG *Mt-dprE2* overexpressor and a strain transformed with the pMV261-empty vector after a 7 day incubation, testing viability with resazurin [21, 27]. The initial screen revealed compounds for which the *Mt-dprE2* overexpressing strain showed a possible increase in the MIC relative to the empty vector strain, and these were further assayed to quantify the MIC using a step gradient of twofold increments of the inhibitor. The secondary screen confirmed that two TB-set compounds, GSK301A and GSK032A, resulted in an eightfold increased MIC when applied to the *Mt*-DprE2 overexpressing strains (Fig. 2). In contrast, overexpression of *Mt*-DprE1 had no systematic effect on the MIC, indicating that these two inhibitors specifically target *Mt*-DprE2 rather than *Mt*-DprE1.

### *Mt*-DprE1 and *Mt*-DprE2 form a complex when co-expressed

**Fig. 3.**
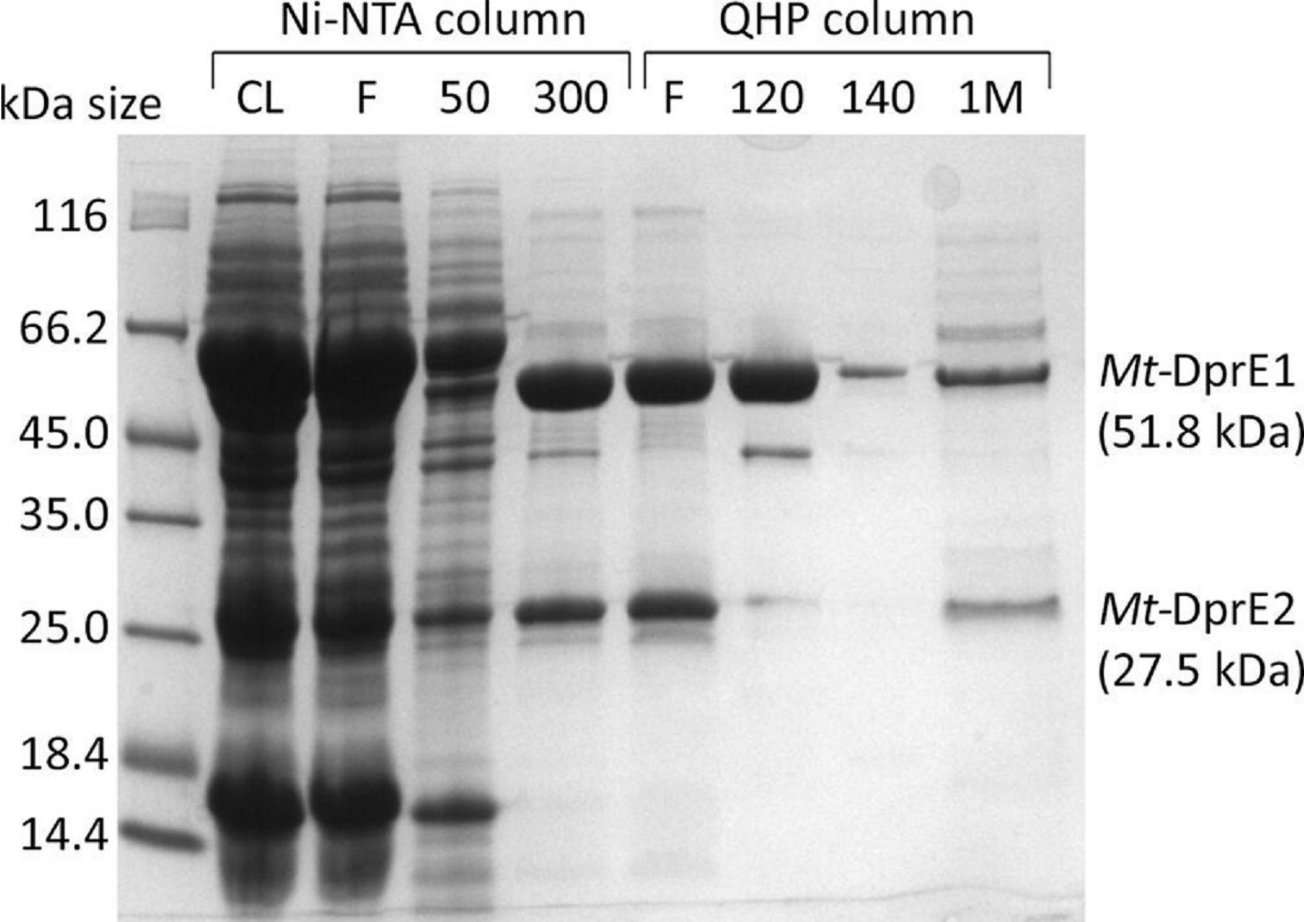
Purification of the *Mt*-DprE1-DprE2 complex. SDS-PAGE of samples of the cleared lysate (CL) and fractions from a nickel-column (Ni-NTA; F=flowthrough and increasing concentrations of imidazole (mM)) and an ion-exchange column [QHP; F=flowthrough and increasing concentrations of NaCl (mM)].

The instability of the keto group in the DPX intermediate suggests that DprE1 and DprE2 might form a complex to ensure loss-free synthesis of the cell-wall precursor DPA. In order to probe complex formation, *Mt*-DprE1 and *Mt*-DprE2 were co-expressed from a pCDF-duet vector (Novagen) in *E. coli* BL21, whereby *Mt*-DprE1 was cloned to include a hexa-histidine tag while *Mt*-DprE2 remained untagged. In addition, the genes were codon optimized for expression in *E. coli* and were co-expressed with the chaperonin combination of *Mt*-Cpn60.2 and *E. coli* GroES, as previously described for *Mt*-DprE1 [20]. Despite lacking the high-affinity tag, *Mt*-DprE2 (27.5 kDa) consistently co-eluted with His_6_-tagged *Mt*-DprE1 (51.8 kDa) from a nickel-NTA column resin at concentrations above 300 mM imidazole (Fig. 3). In addition, the two proteins both flowed straight though a Q-sepharose (QHP) column, whereas *Mt*-DprE1 on its own binds and elutes at 120–140 mM NaCl. In the co-elution fraction, both proteins appeared to be in approximately equimolar amounts as judged by band intensity on the Coomassie-stained SDS-PAGE gel (Fig. 3).

### *Mt*-DprE2 relieves substrate inhibition of *Mt*-DprE1 and amplifies *Mt*-DprE1 activity

**Fig. 4.**
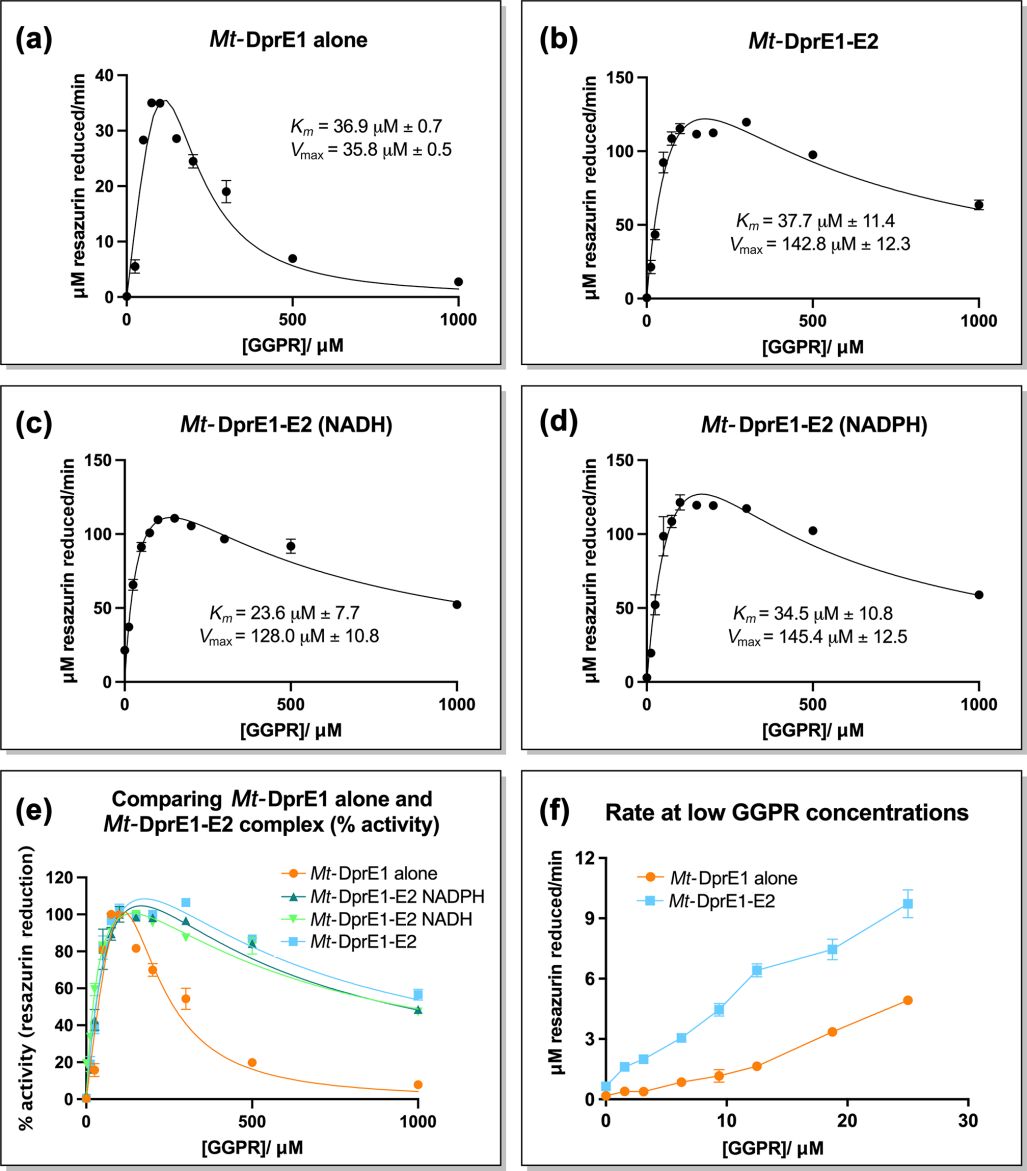
*Mt-*DprE1 assay. A plot of velocity (resazurin reduction µM min^−1^) vs. substrate concentration (GGPR). (a) *Mt*-DprE1 alone and (b)*, Mt*-DprE1-DprE2 complex. The complex was additionally assayed in the presence of (c), NADH (100 µM) and (d), NADPH (100 µM), (e) a comparison of all the DprE1 assay graphs for % activity and (f) a comparison of velocity for *Mt*-DprE1 alone and *Mt*-DprE1-E2 complex at low GGPR concentrations. Values of *K_m_* and *V*_max_ were calculated using Prism GraphPad for sub-inhibitory substrate concentrations.

In order to assess whether the presence of *Mt*-DprE2 and potential complex formation with *Mt*-DprE1 affects the enzymatic activity of the latter, we performed a DprE1 assay as described previously [21], titrating the DPR substrate analogue, geranylgeranylphosphoryl-β-d-ribose (GGPR) (Fig. 4, Table 1). Initial comparisons between *Mt*-DprE1 alone and the *Mt*-DprE1-DprE2 complex (Fig. 4a, b and e) showed that *Mt*-DprE1 substrate turnover is enhanced in the complex, with a *V*_max_ of 142.8±12.3 µM min^−1^, compared to just over 35.8±0.5 µM min^−1^ for *Mt*-DprE1 alone. As observed previously [21], high substrate concentrations (above 300 µM GGPR) inhibited *Mt*-DprE1 activity, but it was intriguing to observe that in the presence of *Mt*-DprE2, substrate inhibition is much reduced, even at GGPR concentrations of 1 mM, which fully inhibits *Mt*-DprE1 alone. To assess the requirement of the *Mt*-DprE2 reduced nicotinamide adenine dinucleotide cofactor (NADH or NADPH) for the DprE1 assay, the GGPR titration assay was repeated for the *Mt*-DprE1-DprE2 complex in the presence of 100 µM NADH or NADPH (Fig. 4c, d and e). Neither cofactor changed the kinetics of *Mt*-DprE1 activity relative to the absent cofactor, indicating that the cofactor does not appreciably affect the DprE1 assay or the observed increase in *V*_max_ and the reduced substrate inhibition of *Mt*-DprE1. The previously observed [21] sigmoidal behaviour of DprE1 activity at low substrate concentrations (GGPR) was absent when *Mt*-DprE1 activity was assayed in the presence of *Mt*-DprE2 (Fig. 4f).

**Table 1.** *Mt-*DprE1 and *Mt-*DprE2 assay kinetics data. *K_m_* and *V*_max_ data calculated using Prism GraphPad for sub-inhibitory substrate concentrations. *Mt-*DprE2 assay data for GGPR titration, with NADH or NADPH cofactors, and cofactor titration with constant GGPR at a sub-inhibitory concentration

Assay			*K_m_* (µM)	*V*_max_ (µM min^−1^)
DprE1 (GGPR titration)	*Mt-*DprE1 alone		36.9±0.7	35.8±0.5
	*Mt*-DprE1-E2		37.7±11.4	142.8±12.3
	*Mt-*DprE1-E2 (NADH)		23.6±7.7	128.0±10.8
	*Mt-*DprE1-E2 (NADPH)		34.5±10.8	145.4±12.5
DprE2	GGPR titration (NADH)		38.0±13.7	20.0±1.8
	GGPR titration (NADPH)		270.7±231.1	23.7±12.2
	NADH titration		10.5±5.6	14.3±1.8
	NADPH titration		34.6±11.2	11.2±0.9

### Developing a spectrometric assay to characterize *Mt-*DprE2 *in vitro* activity

*In vitro* enzymatic activity of *Mt*-DprE2 has not previously been characterized in a quantitative fashion, owing to two primary obstacles: firstly, providing the DPX reaction intermediate (or a suitable analogue thereof) and, secondly, preparing purified recombinant *Mt*-DprE2. We have overcome these obstacles by purifying the *Mt*-DprE1-DprE2 complex from *E. coli* cell extracts and feeding the soluble DPR analogue GGPR (geranylgeranylphosphoryl-β-d-ribose) to the reaction mixture. As a measure of activity, the assay tracks the change in fluorescence of NAD(P)H (excitation at 340 nm, emission at 445 nm), as *Mt*-DprE2 activity depletes the reduced form of the redox cofactor [11]. During the reaction, oxidation of NAD(P)H reduces the keto group of the reaction intermediate DPX, represented in this assay by GGPX (geranylgeranylphosphoryl-β-D-2′-keto-erythro-pentafuranose), to a hydroxyl group, resulting in GGPA.

In initial assays, we titrated the *Mt*-DprE1-DprE2 complex to a reaction mixture containing 200 µM GGPR and 100 µM NADH or NADPH, up to a maximum concentration of 40 µM of the complex, resulting in a concomitant increase of the rate of NADH and NADPH oxidation (Fig. S1a). In contrast, titrating *Mt*-DprE1 alone had little effect: *Mt*-DprE1 at a concentration of up to 30 µM increased oxidation of the cofactors only marginally, whereas a much more drastic effect was produced by the *Mt*-DprE1-DprE2 complex in the concentration range of 10 to 40 µM (Fig. S1a, available in the online version of this article). The new assay was further examined using BTZ as an inhibitor of DprE1 (Fig. S1b). Inhibition of *Mt*-DprE1 (Fig. S1bi) and the *Mt*-DprE1-DprE2 complex (Fig. S1bii) was determined using the DprE1 assay and was additionally measured for the complex using the DprE2 assay with NADH and NADPH (Fig. S1biii). All assays generated similar inhibition curves, with IC_50_ values within a threefold range (5.7±0.5 µM, 13.1±1.1 µM, 4.1±0.5 µM and 5.6±1.1 µM, respectively). The observed inhibition in the DprE2 assay by BTZ, a validated, covalent DprE1 inhibitor, demonstrates that DprE1 activity is required (to produce the reaction intermediate). These data indicate that the assay is sensitive enough to detect *in vitro* enzyme inhibition.

The validated assay was then used to probe inhibition of *Mt*-DprE2 activity by the two compounds from the GSK TB-set, GSK301A and GSK032A, identified as targeting *Mt-*DprE2 in the overexpression assay. However, neither compound showed an inhibitory effect, even when inhibitor and enzyme were pre-incubated with a variety of combinations of substrates (data not shown), suggesting that the differential effect of GSK inhibitors GSK301A and GSK032A on culture growth of *Mt*-DprE2 overexpressors versus *in vitro* Mt-DprE2 activity could indicate a requirement for metabolic conversion from pro-drug to active drug forms.

### Substrate inhibition of *Mt*-DprE2 activity

**Fig. 5.**
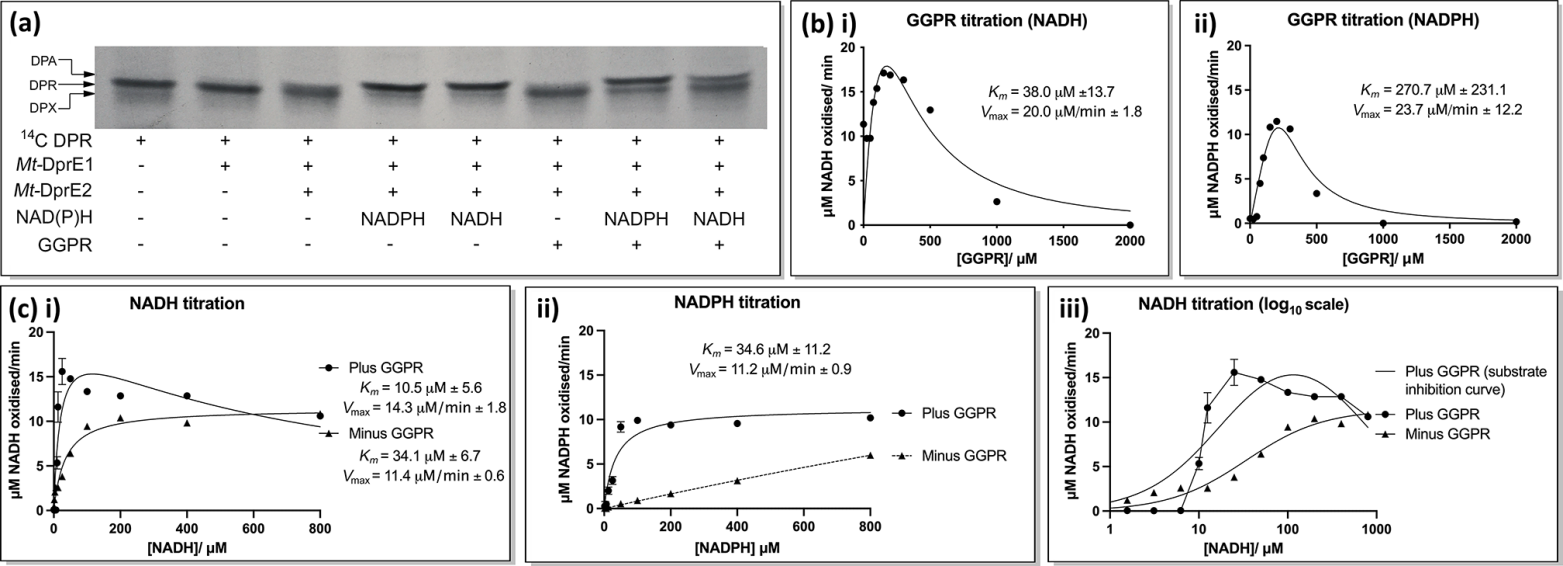
*Mt-*DprE2 assay. (a) TLC of a radioactive assay, using ^14^C-labelled DPR as a substrate. 2000 cpm of ^14^C-DPR was incubated for 1 h at 37 °C in the presence of combinations of 10 µM *Mt*-DprE1 alone, 10 µM *Mt*-DprE1-DprE2 complex, 100 µM NADH/NADPH and 200 µM cold GGPR. (b) Plot of velocity vs. GGPR concentration using cofactors (i) NADH and (ii) NADPH. (c) Plot of velocity vs. cofactor concentration for (i) NADH and (ii) NADPH. (iii) velocity vs. NADH concentration plotted log_10_-scale. Values of *K_m_* and *V*_max_ were calculated using Prism GraphPad for sub-inhibitory substrate concentrations.

The requirement for the reduced nicotinamide adenine dinucleotide cofactor was verified using the radioactive substrate ^14^C-DPR, separating products by TLC (Fig. 5a). In the absence of a cofactor, the intermediate DPX is present, whereas both NADH and NADPH are utilized by *Mt*-DprE2 to reduce DPX to the DPA product. Cold GGPR, used to increase the substrate concentration, also demonstrated that *Mt*-DprE2 can take either cofactor, though NADPH appeared to have a slightly better turnover in this assay. Titrating the cofactors in the DprE2 assay also demonstrated that *Mt*-DprE2 can use either form to reduce DPX, though there was a threefold lower apparent *K_m_* for NADH (10.5±5.6 µM) than for NADPH (34.6±11.2 µM) (Fig. 5bi, ii; Table 1). Interestingly, concentrations of NADH (greater than 25 µM) mildly inhibit the activity of the complex, whilst NADPH showed no such inhibition, even at concentrations of up to 800 µM.

Substrate inhibition by GGPR was apparent not only when assaying *Mt*-DprE1 activity, but also when probing activity of the *Mt*-DprE1-E2 complex in the DprE2 assay (Fig. 5, Table 1). Inhibition was more pronounced in assays that used NADPH rather than NADH as a cofactor, beginning at GGPR concentrations above 350 µM, with the velocity decreasing quickly to almost to zero at a substrate concentration of 1 mM (Fig. 5ci and ii). When using the cofactor NADH, substrate inhibition was also apparent at concentrations above 350 µM GGPR, but concentrations of 2 mM GGPR were required to reduce activity to baseline level. The presence of each cofactor also had a distinct effect on the affinity of the complex for the GGPR substrate: the apparent *K_m_* was over sevenfold lower in the presence of NADH compared to NADPH (38.0±13.7 µM vs. 270.7±231.1 µM).

*Mt*-DprE2 was also found to turn over NADH, with Michaelis–Menten kinetics, in the absence of the substrate, GGPR (Fig. 5ci and iii). This activity was observed at concentrations as low as 1.5 µM NADH, though the apparent *K_m_* was higher (34.1±6.7 µM cf. 10.5±5.6 µM). Conversely, in the presence of GGPR, NADH oxidation was not observed until a concentration of 10 µM NADH was reached. The kinetics of NADH oxidation in the presence and absence of the substrate are quite different and indicate that the presence of the GGPR substrate alters the kinetic activity of the *Mt*-DprE2 enzyme. Turnover of NADPH by *Mt*-DprE2 was not observed in the absence of GGPR, though some spontaneous oxidation was evident at higher NADPH concentrations (Fig. 5cii).

### Generation of spontaneous resistance mutants suggests an activation pathway for GSK301A and GSK032A

In an attempt to determine a potential pro-drug activation pathway for the GSK compounds, spontaneous resistant mutants were generated to GSK032A and a derivative, GW432086X, using *M. bovis* BCG strain. One mutant grew for each compound, both with fivefold higher MICs compared to the WT parental strain, and cross-resistance to compound derivatives. The genomes of these two resistant mutants were isolated and sequenced using whole-genome sequencing (WGS). These sequences were aligned to the parental strain, to establish the genes responsible for the observed resistance. This identified two genes, *fgd1* (S196P) and *fbiC* (R847H), for GSK032A and GW432086X, respectively. This method did not identify any resistant mutations in *dprE2* and could be indicative of the activation pathway for these compounds.

### Generating a structural model of the *Mt*-DprE1-E2 complex

In order to rationalize the effects of complex formation seen in the kinetic analyses, we constructed a model of the *Mt*-DprE1-DprE2 complex using AlphaFold2. A series of crystallographic structures are available for *Mt*-DprE1, but the tendency of *Mt*-DprE2 to form aggregates after purification has so far prevented its crystallization or of the *Mt*-DprE1-DprE2 complex. In AlphaFold2, we co-folded the sequences of the two proteins and explored the effect of sequence variation on the resulting model by repeating the *in silico* folding with the sequences of several DprE1/E2 orthologues. The predicted structure of *Mt*-DprE1 is near identical to representatives of the crystallographic structures (RMSD ~0.7 Å for aligned 404 Cα atoms, reference structure pdb 4kw5), while the model of DprE2 matches with an RMSD of ~1.3 Å to the structure of the NADPH-dependent reductase PhaB of *Ralstonia eutropha* (pdb entry 3vzs, 29 % sequence identity [28]), its closest sequence homologue of known structure.

**Fig. 6.**
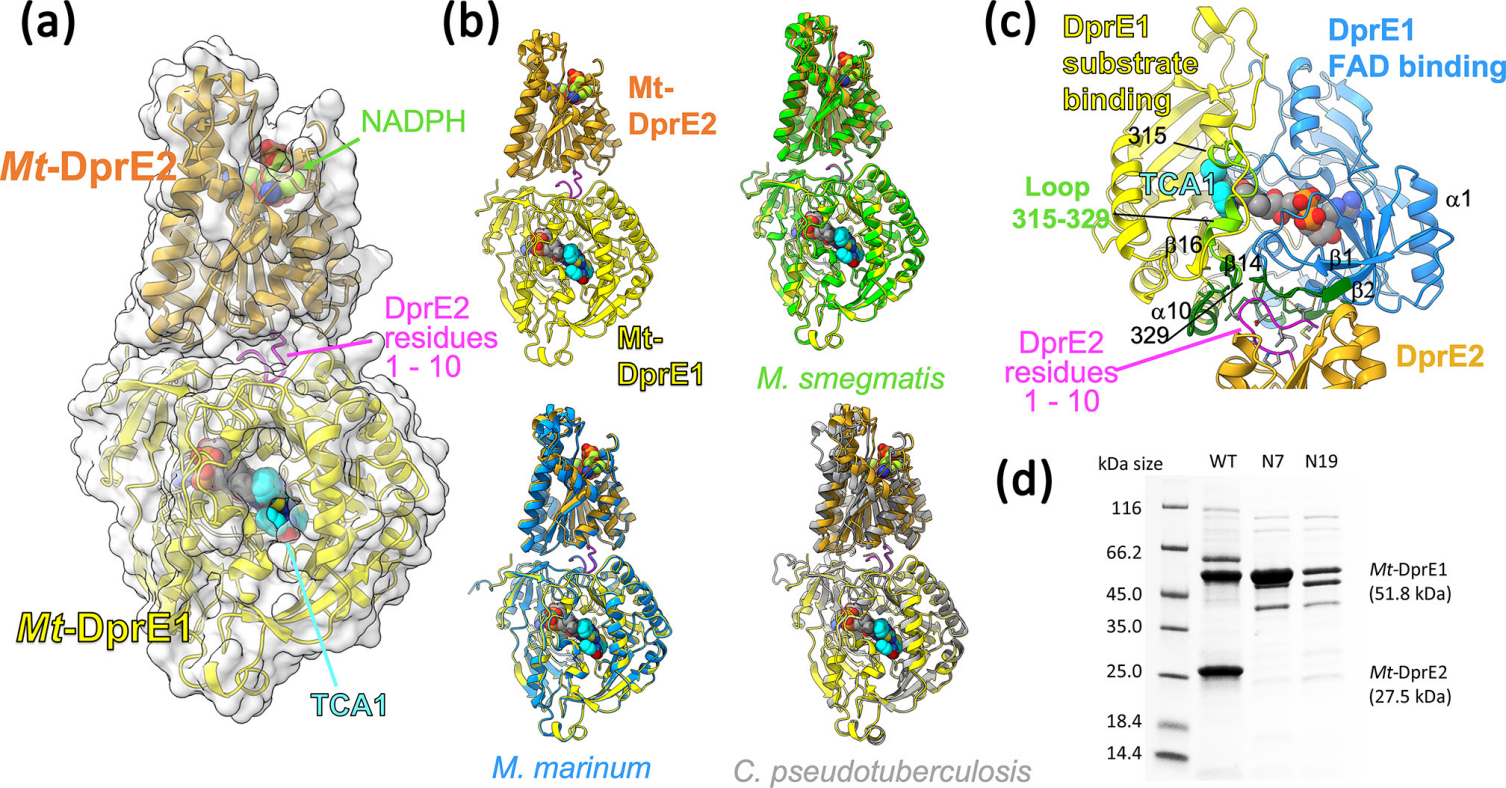
Structural model for the *Mtb* DprE1-DprE2 complex generated with AlphaFold2. (a) Overview of the predicted complex structure, with proteins represented as ribbon diagrams and with translucent molecular surfaces. DprE1 is shown with FAD (grey spheres) and the inhibitor TCA1 (spheres in cyan) according to the superposition with the coordinates of pdb entry 4kw5 (PMID 23776209). NADPH was docked into the structure of DprE2 according to the structural superposition with *Ralstonia eutropha* PhaB (pdb entry 3vzs, PMID 23913421). (b) Superposition of the predicted structures for orthologues from *M. smegmatis* (green ribbon), *M. marinum* (light blue) and *Corynebacterium pseudotuberculosis* (grey ribbon). (c) Close-up view of the DprE1:DprE2 interface, with DprE1 residues buried upon complex formation shown in green. Selected secondary structure elements of DprE1 are labelled according to nomenclature used in PMID 22733761. Spheres in cyan represent the inhibitor TCA1 (structural superposition with pdb entry 4kw5, PMID 23776209). (d) SDS-PAGE comparing the *Mt*-DprE1-DprE2 complex WT (full length *Mt*-DprE2) with the N-terminally truncated *Mt*-DprE2, N7 and N19 (beginning on the seventh and nineteenth amino acid, respectively). All samples were eluted from a Ni-NTA column resin at 300 mM imidazole.

The predicted structural models of the DprE1-E2 complex for the sequences of *Mtb*, *M. smegmatis *, *Mycobacterium marinum* and *Corynebacterium pseudotuberculosis *, which share between 61 and 91% sequence identity for DprE2 (Table S1), are remarkably consistent. Common to these predicted complexes is that the N-terminal sequence of DprE2 (residues 1–10) folds into a turn, which docks into a surface conserved residues on DprE1 (Fig. 6, Fig. S2a). In contrast, the prediction of the *Pseudomonas aeruginosa* orthologues suggests a different complex interface (Fig. S2b). The highly conserved N-terminal tail seen in *Mt*-DprE2 is missing in the *P. aeruginosa* sequence (Fig. S2c), which may explain why the prediction for the *P. aeruginosa* sequences does not match the other four.

We have sought to verify the predicted complex by purifying complexes where the N-terminal sequence of DprE2 was truncated by 6 and 18 residues, respectively (mutants DprE2-N7, DprE2-N19). Gel electrophoresis clearly demonstrates that His-tagged DprE1 binds the untagged mutants DprE2-N7, DprE2-N19 far less efficiently than wild-type DprE2 (Fig. 6d). Similarly, analysing fractions eluted from the Ni-NTA chromatography column demonstrates that the truncated (untagged) DprE2 species are lost early during the imidazole concentration gradient (Fig. S2d), whereas wild-type (untagged) DprE2 co-elutes with the His-tagged DprE1 (Fig. 3).

In the predicted structures the active sites of DprE1 and DprE2 appear on opposite surfaces of the complex (Fig. 6a), suggesting that no substrate channelling occurs between the partner enzymes. The active site of DprE2 is situated diametrically opposite to the predicted complex interface, while the margins of the active site of DprE1 overlap with parts of the DprE1/E2 interface (Fig. 6c). Residues at the C-terminal end of the disordered active site loop (residues 315 to 329) form part of the interface as do residues in the β1-β2 loop (residues 19–23) (Fig. 6c, Fig. S2a), where the tip of the β1-β2 loop (Trp16) is in close contact with the isoalloxazine rings of the flavin cofactor. A third element of the DrpE1:E2 interface are residues 396, 397 in the β16-α10 loop, wherein strand β16 is part of the ‘floor’ of the substrate-binding site of DprE1.

## Discussion

Overcoming the resistance of *M. tuberculosis* to antibiotics used in TB therapy is a key strategy in achieving WHO’s ambitious efforts to contain if not eradicate tuberculosis world-wide. This requires identifying novel compounds with antitubercular activity that hit as yet unexploited cellular targets. Here, we were able to demonstrate that *Mt*-DprE2 is a useful target for the development of new antibiotics against *Mtb*. Two compounds GSK301A and GSK032A from the GSK 177 TB-set were found to target *Mt*-DprE2 based on elevated MICs in overexpressing strains of *M. bovis* BCG. Secondly, by using the purified *Mt*-DprE1-DprE2 complex, we were able to mitigate *Mt*-DprE2 aggregation, enabling us to establish a spectroscopic assay that probes *Mt*-DprE2 activity by tracking oxidation of the cofactor NAD(P)H. This advance supports future library screens for compounds that target DprE2. While the growth-inhibiting activity of GSK301A and GSK032A did not translate to measurable inhibition of *Mt*-DprE2 activity *in vitro*, we were able to demonstrate that our DprE2 assay responds to small-molecule inhibition of *Mt*-DprE1 activity in a quantitative fashion.

**Fig. 7.**
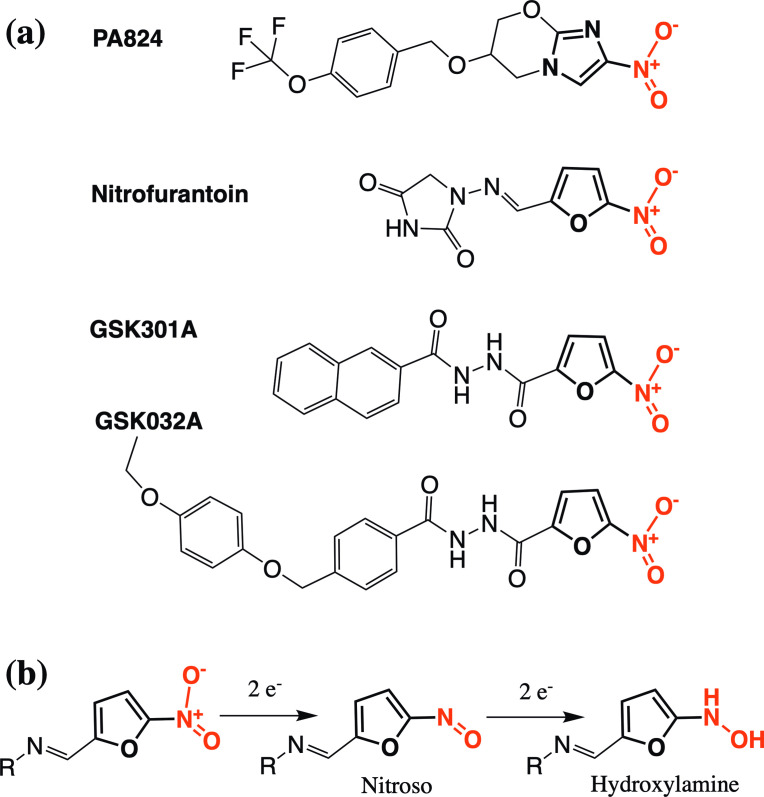
Comparison of the structures of GSK301A, GSK032A, nitrofurantoin and PA824. (a) Structures of compounds: the common nitro group is red, while the adjoining ring is bold (furan for GSK301A, GSK032A and nitrofurantoin; imidazole ring for PA824). (b) Suggested activation pathway of nitrofurantoin [41, 42].

Lack of inhibitory capacity in our *in vitro* assay could indicate that both compounds, which have similar structures and potently inhibit growth in culture, first require metabolic conversion to an active form. Pro-drug activation is a feature in other nitro-compounds. For example, the BTZ class of anti-tubercular inhibitors possess a nitro group that is reduced to a nitroso group before the inhibitor can form a covalent complex with DprE1. In this case, the activating enzyme is identical to the inhibitor target in that activation of BTZ compounds is achieved by the reduced flavin cofactor of DprE1, after a single turnover of substrate [20, 29–31]. Spontaneous mutants, resistant to the GSK compounds, had mutations in *fgd1* and *fbiC*, genes that are part of the well-known activation pathway of the pro-drugs, pretomanid [32, 33] and delamanid [34]. This pathway also includes the dezaflavin (F_420_)-dependent nitroreductase, Ddn, which activates these compounds by reducing the aromatic nitro group (Fig. 7) [35, 36]. FbiC forms part of the F_420_ synthesis pathway [37], while the activity of Fgd1, glucose-6-phosphate dehydrogenase, acts in the redox cycling of the cofactor to the reduced state required by Ddn [35]. The discovery of other pathways through the generation of resistant mutants is not unique and has also been used to determine the activation mechanism of the first-line (pro-) drug isoniazid, which is activated by catalase peroxidase KatG and inhibits 2-trans-enoyl-acyl carrier protein reductase, InhA, of the fatty acid synthase II system [38].

Interestingly, GSK301A and GSK032A have similar structures, sharing a common nitro group on a furan ring, a feature that is also part of the antibiotic nitrofurantoin (Fig. 7a). While the exact mechanism of action of nitrofurantoin is not clear, similarly to the GSK compounds, activation occurs through the activity of bacterial nitroreductase flavoproteins [39]. The nitro group is reduced to nitroso and hydroxylamine species, one or both of which inhibit enzymes involved in DNA/RNA and protein synthesis (Fig. 7b) [40–42]. Whilst the aromatic nitro group of PA824 has an imidazole in place of the furan ring, the shared activation pathway with GSK301A and GSK032A and structural similarities of the GSK compounds with nitrofurantoin, it seems reasonable to speculate that activation of these compounds could all proceed through a similar reduction of the nitro group. The active form of PA824 is not currently known and when reduced experimentally by purified Ddn or in *Mtb*, a number of metabolites are produced, many of which are short lived, though a nitroso to hydroxylamine pathway is not amongst those currently predicted [43].

It had been previously suggested that DprE1 and DprE2 form a complex, and we have been able to confirm complex formation, as the two enzymes co-purified through successive Ni-NTA and ion exchange chromatography steps, whereby *Mt*-DprE2 did not carry a multi-histidine tag. The complex is active as demonstrated by the DprE1 and DprE2 assays, and it mitigates (but does not solve) the problem of *Mt*-DprE2 tending to aggregate.

The assay for *Mt*-DprE1 shows a sigmoidal saturation curve with respect to substrate concentrations [21], a feature that was replicated in this study. Sigmoidal enzyme kinetics normally indicates cooperativity, for instance, between identical subunits of a multimeric enzyme or between alternative binding sites on the same subunit. Our previous analyses have shown *Mt*-DprE1 consistently as a monomer [20] and the presence of a single flavin structure in complex, with various inhibitors only indicated a single binding site. Still, sigmoidal kinetics of monomeric, single-site enzymes has been observed previously, including human glucokinase [44] and alternative models for how cooperativity effects can emerge in such enzymes have been proposed [44]. Nevertheless, the sigmoidal kinetics is not observed for the *Mt*-DprE1-DprE2 complex, at least not when using GGPR as a substrate analogue. It is possible that sigmoidal kinetics is an artefact of assaying *Mt*-DprE1 in isolation, whereas *in vivo* it may work predominantly as part of the *Mt*-DprE1-DprE2 complex. Substrate channelling within the 2-enzyme complex would be a mechanistically plausible way to ensure the efficient conversion of DPR to DPA without exposing the reaction intermediate DPX to the cellular environment.

The presence of *Mt*-DprE2 relieved the apparent substrate inhibition observed in DprE1 activity assays [21], a feature that may also explain the increase in turnover of the complex compared to *Mt*-DprE1 alone. We previously interpreted apparent substrate inhibition at higher GGPR concentrations as the result of a detergent-like effect of the substrate’s geranylgeranyl fatty acid chain. However, CD spectra (data not shown) indicated that GGPR had no quantifiable effect on the secondary structure of the complex, even at high concentrations. Therefore, the ability of *Mt*-DprE2 to relieve substrate inhibition of *Mt*-DprE1 could indicate that the observed inhibitory effect of high GGPR concentration on *Mt*-DprE1 activity may rather be a consequence of slow release of the *Mt*-DprE1 product, GGPX. In the context of the *Mt*-DprE1-DprE1 complex, release of the reaction intermediate GGPX to *Mt*-DprE2 may result in the efficient conversion to the final product, GGPA. Nevertheless, apparent substrate inhibition at high GGPR concentration re-appears when assaying *Mt*-DprE2 activity, perhaps once more reflecting inefficient release of product rather than actual substrate inhibition.

Interestingly, when titrating GGPR in the DprE2 assay, there was a difference in the kinetics of NADH oxidation versus NADPH oxidation. Both cofactors have been suggested for DprE2, though the preference is not known. In our assays, although *Mt*-DprE2 had a much higher affinity for NADH, higher concentrations mildly inhibited the activity of the complex, while there was no such inhibition indicated for NADPH. NADH also enhanced the affinity of *Mt*-DprE2 for the GGPR substrate. In contrast, the presence of NADPH increased the effect of substrate inhibition at higher GGPR concentrations, when compared to NADH. Overall, the data would suggest that NADH is the natural cofactor, with higher affinities observed for this cofactor and GGPR substrate; although if a tighter substrate/product inhibition mechanism is desired, then perhaps NADPH would be the more likely cofactor. DPA, the natural product of the DprE1-DprE2 complex is essential to *Mtb*, not only for the synthesis of the arabinan domains of the AG and LAM, but also as a pool of decaprenyl phosphate, which is a critical base for other lipid-anchored substrates, including lipid II in peptidoglycan synthesis [45] and decaprenyl phosphomannose in lipomannan and lipoarabinomannan synthesis [46]. Therefore, it could be speculated that tight regulation of this molecule is crucial to *Mtb* and as such tight substrate/product inhibition of the enzymes in the biosynthesis pathway would be a necessary feature. While to many, substrate inhibition is a consequence of the unusually high substrate concentrations used an *in vitro* setting, it is also mechanism that is widely used by many enzymes for feed-back regulation [47]. Substrate inhibition is known to be crucial to several processes in mammals, not only as a mechanism that ensures a product is only synthesized as required, such as ATP in glycolysis [47], but also a way of maintaining the levels of critical compounds that have fluctuating substrate concentrations, such as dopamine in the brain [48]. A gluco-oligosaccharide oxidase from the fungus *Sarocladium strictum*, which similarly to DprE1 possesses a FAD cofactor, also shows substrate inhibition; while the biological significance of the inhibition was not studied, it was found to be reduced by amino acid substitutions in the substrate binding site [49]. Less is known of the significance of substrate inhibition to prokaryotes, although many prokaryotic enzymes have this property, such as the nitric-oxide reductase from *Paracoccus denitrificans* [50].

The prediction of the complex structure of Mt-DprE1-DprE2 by the AlphaFold2 algorithm evidently needs experimental confirmation. For DprE1, the predicted fold is astonishingly close to the known experimental structures, while for DprE2 the prediction matches crystallographic structures of non-mycobacterial homologues. The involvement of the N-terminal sequence of *Mt*-DprE2 in complex formation is experimentally supported by observing abrogation of complexation when the N-terminal 6 or 18 residues of DprE2 are truncated (Fig. 6d). In contrast, AlphaFold2 predicted a different complex interface for *P. aeruginosa *, a species in which the N terminus of DprE2 lacks eight residues relative to *Mt-*DprE2 (Fig. S2b). Secondly, the predicted structure provides mechanistic hints for why complex formation with DprE2 might affect the kinetic behaviour of *Mt*-DprE1. The complex interface is adjacent to the active site of DprE1, and non-covalent contacts involve structural elements connected to the DprE1 active site (Fig. 6c). For instance, contacts to DprE1 residues 330–333 will likely affect the ordering of the loop spanning residues 315–329 (Fig. 6c). This loop is disordered in a subset of crystallographic structures, but in the ordered state occludes the active site, sterically hindering substrate binding and product release. Similarly, complex formation involves residues in the β1-β2 loop, which makes contacts with the redox-active isoalloxazine moiety of FAD, likely influencing the kinetic behaviour of DprE1. Thus, the predicted location of the complex interface relative to the active site of DprE1 rationalizes potential allosteric effects of DprE2 binding on the reaction kinetics of DprE1.

In conclusion, this study has provided the initial groundwork for a new drug target for *Mtb*, namely DprE2. This includes the identification of two putative inhibitors from the GSK TB-set and the development of a new spectrophometric assay for DprE2 activity, which will pave the way to the identification of more inhibitors using drug libraries. The purification of the *Mt*-DprE1-DprE2 complex could also be useful for structural studies, although the observed *Mt*-DprE2 aggregation makes this problematic and will require more research. With the surge in resistant strains, it is ever more important that new targets, such as DprE2, are studied, in the hope that new and clinically significant antibiotics can be identified and further developed.

## Experimental procedures

### Cloning *Mt-dprE1* and *Mt-dprE2* into pMV261 and pCDF-duet

*Mt-dprE1* (Rv3790) was cloned into pMV261 as previously described [21]. *Mt-dprE2* (Rv3791) was amplified and cloned using the following primers, ctagctagggatccaatggttcttgatgccgtagg and ctagctagaagctttcagatgggcagcttgcggaag, where the underlined sites introduced compatible restriction sites, *BamHI* and *HindIII*, at the ends. pCDF-duet was produced using codon-optimized genes, for expression in *E. coli. Mt-dprE1* was cloned into the first multiple cloning site, using the primers catgcatgggatccgatgctgtctgttggtgctac and catgcatgaagctttcacagcagttccagacgac, which introduced *BamHI* and *HindIII* into the ends of the amplified gene. *Mt-dprE2* was cloned into the second cloning site, using the primers, catgcatgcatatggtcctggatgctgtgggc and catgcatgctcgagtcaaatcggcagtttacgg, which introduced a stop codon at the end of the coding sequence of the gene and the restriction sites, *NdeI* and *XhoI*. In this configuration, *Mt*-DprE1 is expressed with an N-terminal hexa-his tag, while *Mt*-DprE2 is untagged. The N-terminal truncation derivatives of *Mt*-DprE2 were cloned into the second site of the pCDF-duet-*Mt-dprE1* vector, using the reverse primer as previously, and catgcatgcatatgggcaacccgcaaacggtcc for *Mt*-DprE2 beginning on the seventh amino acid, and catgcatgcatatgagcgaaatcggtctggcaatc for *Mt*-DprE2 beginning on the nineteenth amino acid.

### Whole-cell target-gene overexpression screen

These assays were performed as previously described [21]. Briefly, the TB-set plates were diluted to 1 mM and 0.1 mM with DMSO in 96-well v-shaped polystyrene plates and 2 µl of these intermediate plates was transferred into Greiner black bottom assay plates. *M. bovis* BCG cells harbouring pMV261-empty vector, pMV261-*Mt-dprE1* and pMV261-*Mt-dprE2* were grown to mid-log phase (OD_600_=0.4–0.8) at 37 °C in 7H9 (Middlebrook), supplemented with 10 % (v/v) ADC (Middlebrook), 0.25 % glycerol, 0.05 % Tween-80 and 25 µg ml^−1^ kanamycin, and diluted to 1.5×10^6^ c.f.u. ml^−1^ in the same growth media. Diluted cells (98 µl) were added to the replicated assay plates, which were then sealed with parafilm and incubated at 37 °C in a static, humid, CO_2_ incubator for 7 days. After incubation, 30 µl of 0.02 % (w/v) resazurin and 12.5 µl of 20 % (v/v) Tween-80 was added and the plates incubated for a further 24 h as previously described [21, 27]. The resazurin is reduced to resorufin by viable cells, and the fluorescence intensity of which was measured using a POLARstar Omega plate reader (BMG Labtech), with an excitation at 530 nm and emission at 590 nm. Inhibitors that demonstrated a possible change in the MIC between the pMV261-empty vector and *Mt-dprE1* and *Mt-dprE2* overexpressor strains were serially diluted twofold, at 50× the desired concentrations to cover the MIC, and re-examined in the assay plates using the overexpressor and empty vector harbouring strains.

### Spontaneous resistance mutant generation

This was carried out by serial passage [51], with plating using the 24-well SPOTi assay technique [52]. 24-well compound plates (corning) were set up with 1 ml of 7H10 solid agar, supplemented with 10 % (v/v) OADC, 0.5 % glycerol, 0.025 % Tween-80 and a dilution of the compound, which was serially diluted by 1.25–1.5-fold in DMSO, to cover the MIC and up to 5×the MIC. WT *M. bovis* BCG was grown to mid-log phase (OD_600_=0.4–0.8) at 37 °C in 7H9 (Middlebrook), supplemented with 10 % (v/v) ADC (Middlebrook), 0.25 % glycerol and 0.05 % Tween-80. Next, 2 µl culture was spotted onto each well of the 24-well compound plates, which were then wrapped in parafilm and foil and incubated at 37 °C in a static, humid, CO_2_ incubator, until cell growth appeared. Cells growing on the highest concentration of compound were inoculated to liquid 7H9 broth with no compound as previously, grown to mid-log phase and spotted onto fresh 24-well compound plates. This was repeated until mutants grew at higher compound concentrations. The mutations conferring resistance were determined by whole-genome sequencing (WGS). Genomic DNA was extracted from a 50 ml log-phase culture of the resistant mutant, grown in 7H9 broth without selection, according to standard procedures. WGS was performed by MicrobesNG, along with the alignment to the reference genome, *M. bovis* BCG Pasteur 1173P2 (accession number: NC_008768.1), for the identification of single polynucleotide polymorphisms (SNPs).

### Overexpression and purification of the *Mt*-DprE1-DprE2 complex

The *Mt-*DprE1-DprE2 complex, was overexpressed and purified as for *Mt*-DprE1 alone [20], for both the full length and truncated *Mt*-DprE2. The complex was overexpressed from the pCDF-duet vector in *E. coli* BL21 (DE3), along with the chaperones from the plasmid pTrc-60.2-GroES. Cells were grown at 37 °C in terrific broth (Difco), supplemented with spectinomycin (100 µg ml^−1^) and ampicillin (100 µg ml^−1^), to mid-log phase. At this point, the cells were cooled to 20 °C in an ice bath, before adding 0.5 mM isopropylthiogalactoside (IPTG) and incubating overnight at 20 °C. Cells were pelleted, washed in 0.85 % saline and frozen. Cells were thawed, resuspended in 50 mM Tris-HCL (pH 8), 50 mM NaCl and 10 % glycerol (buffer A), and lysed by sonication (Sonicator Ultrasonic Liquid Processor XL; Misonix) on ice for 10 min (20 s on, with 40 s cooling). The insoluble fraction was separated by centrifugation (40 min, 4 °C and 27 000 ***g***) and the cleared lysate loaded onto a pre-equilibrated 1 ml His-trap column (GE Healthcare). The column was washed with 20 mM imidazole and eluted in 300 mM imidazole, in buffer A. The eluant was loaded onto a pre-equilibrated QHP column (GE Healthcare) and washed/eluted with a gradient of 100–160 mM NaCl in buffer A. The *Mt*-DprE1-DprE2 complex does not bind to the QHP column and passes straight through. *Mt*-DprE1 alone was purified as for the complex, although elutes from the QHP column at 120–140 mM NaCl. The samples were analysed by SDS-PAGE and fractions containing pure proteins pooled and dialysed against buffer A with 200 mM NaCl.

### *Mt*-DprE1 assay

This is a resazurin redox indicator assay as previously described [21], using GGPR as the substrate. The progression of the reaction was measured, through the subsequent reduction of resazurin to resorufin, using a POLARstar Omega plate reader (BMG Labtech) at 37 °C, with excitation at 530 nm and emission at 590 nm. Greiner 384 black-bottom plates were used to set up assays with a 25 µl total volume, containing 50 mM Hepes, pH 7.5, 100 mM NaCl, 2 µM FAD and 100 µM resazurin, with 10 µM *Mt*-DprE1 or 10 µM *Mt*-DprE1-DprE2 and variable concentrations of GGPR. The reaction was initiated through the addition of the enzyme using the injection pump on the plate reader. BTZ inhibition was measured by adding 0.5 µl of a twofold dilution series of BTZ in DMSO at 50× the final concentration, using 200 µM GGPR. Fluorescence was converted to µM resorufin reduced using a resorufin standard curve.

### *Mt*-DprE2 assay

This assay was developed based on the decrease in fluorescence of the DprE2 cofactors, NADH and NADPH, upon oxidation. DprE2 is a reductase and uses the cofactor NAD(P)H to reduce the keto group of the intermediate, in this case GGPX, to a hydroxyl group, which in turn oxidises the cofactor to NAD(P)^+^. Greiner 384 black-bottom plates were used to set up assays with a 25 µl total volume, containing 50 mM Hepes, pH 7.5, 100 mM NaCl, 2 µM FAD, with 10 µM *Mt*-DprE1-DprE2, 200 µM GGPR and 100 µM NADH/NADPH, when fixed concentrations were used. The reduction in fluorescence was measured using a POLARstar Omega plate reader (BMG Labtech) at 37 °C, using excitation at 340 nm and emission at 445 nm. The assay was initiated either by enzyme or GGPR addition using the injection pump on the plate reader. BTZ inhibition was measured by adding 0.5 µl of a twofold dilution series of BTZ in DMSO at 50× the final concentration. Fluorescence was converted to µM NADH/NADPH oxidized using a standard curve.

### Enzyme kinetic analysis

All graphs were drawn using Prism GraphPad. Michaelis−Menten kinetics data was also calculated using Prism. Substrate inhibition curves were plotted using Prism or the Dynafit software and the equations in Scheme 1 [53], where Ks and Kss are dissociation constants and kcat is the turnover of the enzyme in min^−1^. The *K_m_* and *V*_max_ were determined using Prism, using points below the substrate inhibition concentrations.


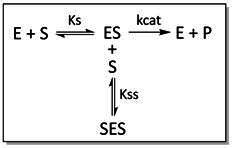


**Scheme 1.** Mechanism for substrate inhibition. This mechanism assumes that enzyme I has two substrate (S) binding sites: a catalytic site for normal catalysis and a second non-catalytic or allosteric binding site. When substrate is bound at both sites (SES), the product (P) is produced at a reduced rate.

### ^14^C-DPR radioactive assays

Radiolabelled ^14^C-DPR was prepared as previously [54]. In total, 2000 cpm of ^14^C-DPR was resuspended in 5.5 µl 1 % (v/v) IGEPAL. Reaction mixtures containing combinations of 10 µM *Mt*-DprE1, 10 µM *Mt*-DprE1-DprE2, 100 µM NADH/NADPH and 200 µM GGPR in a buffer of 50 mM Hepes, pH 7.5 and 100 mM NaCl, were incubated at 37 °C for 1 h. The reactions were quenched with 350 µl of CHCl_3_:CH_3_OH (2 : 1, v/v) and the organic and aqueous layers separated by the addition of 55 µl H_2_O. The bottom, organic phase layer was dried down and resuspended in 10 µl CHCl_3_:CH_3_OH (2 : 1, v/v), spotted onto a high-performance aluminium-backed TLC plate and separated by CHCl_3_:CH_3_OH:NH_4_OH:H_2_O [65 : 25 : 0.5 : 3.6, (v/v)]. Bands were visualized by exposing the TLC to a Kodak X-Omat AR film for a week.

### Structure prediction

The complex structure prediction used the AlphaFold2 algorithm, using the AlphaFold2.ipynb interface with default settings (colab.research.google.com/github/sokrypton/ColabFold/blob/main/AlphaFold2.ipynb, Mirdita M, Schütze K, Moriwaki Y, Heo L, Ovchinnikov S, Steinegger M. ColabFo–d - Making protein folding accessible to all. bioRxiv, 2021). Sequences of DprE1 and DprE2 were retrieved from NCBI (ncbi.nlm.nih.gov). The predicted structures were analysed using ChimeraX and PISA.

## Supplementary Data

Supplementary material 1
