# Supplementary material for: Assay development and inhibition of the Mt-DprE2 essential reductase from Mycobacterium tuberculosis
Source: Microbiology (Reading). 2023 Jan 23;169(1):001288. doi: 10.1099/mic.0.001288 (PMC9993113; doi:10.1099/mic.0.001288)
Supplement: Supplementary material 1 [file mic-169-1288-s001.pdf]

Supplementary Material

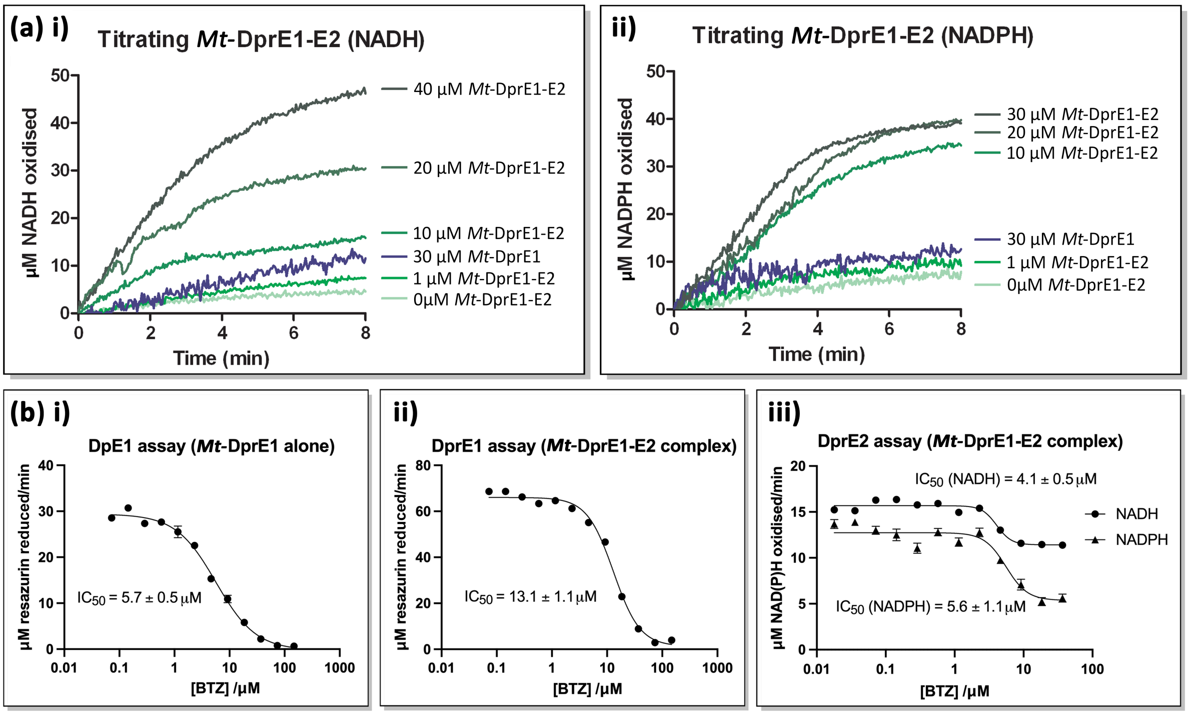

Supplementary Figure S1. *Mt*-DprE2 assay development. Initial raw data to validate DprE2 assay development. a) *Mt*-DprE1-DprE2 complex titration with cofactors i) NADH and ii) NADPH. The assay was performed with varying concentrations of *Mt*-DprE1-DprE2 (labelled), 200 µM GGPR and 100 µM NADH/NADPH, b), Inhibition by BTZ. i) *Mt*-DprE1 alone (DprE1 assay), ii) *Mt*-DprE1-DprE2 complex (DprE1 assay) and iii) *Mt*-DprE1-DprE2 (DprE2 assay). The assay mix contained 10 µM *Mt*-DprE1-DprE2, 200 µM GGPR, 100 µM NADH/NADPH, with varying concentrations of BTZ. Data was plotted and IC_{50} calculated by fitting a four-parameter dose response curve, using Prism GraphPad.

	**Mt_E1**	**Ms_E1**	**Mm_E1**	**Cp_E1**	**Pa_E1**
**Mt_E1**	100	83.48	88.7	70.28	38.06
**Ms_E1**		100	85	72.39	37.12
**Mm_E1**			100	70.63	37.5
**Cp_E1**				100	38.05
**Pa_E1**					100
	Mt_E2	Ms_E2	Mm_E2	Cp_E2	Pa_E2
**Mt_E2**	100	85.04	90.55	60.47	31.15
**Ms_E2**		100	86.61	61.66	30.74
**Mm_E2**			100	61.66	29.51
**Cp_E2**				100	30.74
**Pa_E2**					100

Supplementary Table 1. Sequence identity (in %) of DprE1 and DprE2 (Xx_E2) orthologues after multiple sequence alignment in Clustal Omega (Madeira F et al. (2022), Nucl Acids Res, PMID 35412617). Species names are abbreviated as: Mt – *M. tuberculosis*, Ms – *M. smegmatis*, Mm – *M. marinum*, Cp – *Corynebacterium pseudotuberculosis*, Pa – *Pseudomonas aeruginosa*.

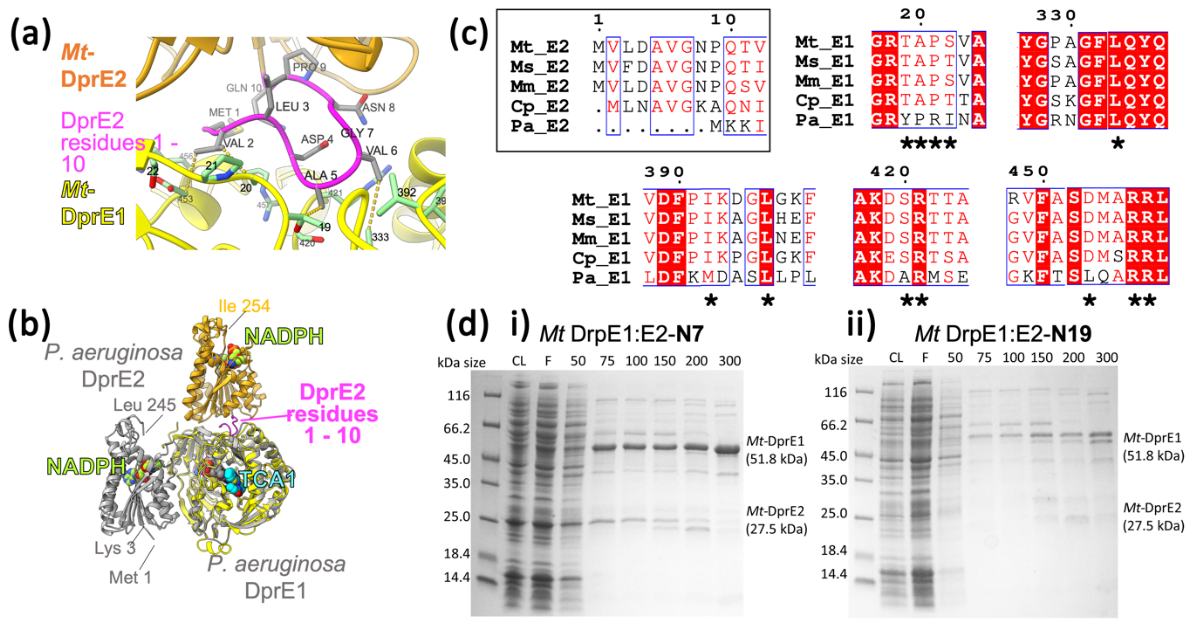

Supplementary Figure S2. Role the N-terminal sequence of DprE2 in complex formation with DprE1. a) *Mt*-DprE1:DprE2 interface with the N-terminal sequence of DprE2 (magenta). Residues in DprE2 are labelled with amino acid type and sequence number, while only residue numbers are given for

DprE1. Selected non-covalent contacts are indicated by dashed lines. b) Superposition of the AlphaFold2-predicted DprE1:DprE2 complex structures for *M. tuberculosis* (ribbons in yellow and orange) and *Pseudomonas aeruginosa* (ribbon in grey). AlphaFold2 predicts a different complex interface for the latter compared to the mycobacterial orthologues. c) Sequence alignments of residues involved in interface formation for DprE2 (boxed) and DprE1. Sequences were aligned using Clustal Omega (PMID 35412617) and formatted using Espript (PMID 24753421); asterisks indicate residues of Mt-DprE1 in contact with the N-terminal sequence of DprE2; species names are abbreviated as Mt – *M. tuberculosis*, Ms – *M. smegmatis*, Mm – *M. marinum*, Cp – *Corynebacterium pseudotuberculosis*, Pa – *Pseudomonas aeruginosa*. d) SDS-PAGE of the Ni-NTA purification of the *Mt*-DprE1-DprE2 complex with N-terminally truncated *Mt*-DprE2, N7 and N19 (beginning on the 7th and 19th amino acid respectively). Labels across the top are: CL = cleared lysate; F = flowthrough; imidazole concentrations given in mM.
